# Effect of aortic valve replacement on myocardial perfusion and exercise capacity in patients with severe aortic stenosis

**DOI:** 10.1038/s41598-024-72480-2

**Published:** 2024-09-14

**Authors:** Saadia Aslam, Abhishek Dattani, Aseel Alfuhied, Gaurav S. Gulsin, Jayanth R. Arnold, Christopher D. Steadman, Michael Jerosch-Herold, Hui Xue, Peter Kellman, Gerry P. McCann, Anvesha Singh

**Affiliations:** 1 Department of Cardiovascular Sciences, University of Leicester and the NIHR Leicester Biomedical Research Centre, Glenfield Hospital, Leicester, UK; 2 Department of Cardiovascular Technology – Echocardiography, College of Applied Medical Sciences, King Saud Bin Abdulaziz University for Health Sciences, Riyadh, Kingdom of Saudi Arabia; 3 Poole Hospital NHS Foundation Trust, Poole, UK; 4 Department of Radiology, Brigham and Women’s Hospital and Harvard Medical School, Boston, MA USA; 5 National Heart, Lung, and Blood Institute, National Institutes of Health, Bethesda, USA

**Keywords:** Myocardial perfusion reserve, Exercise capacity, Aortic stenosis, Aortic valve replacement, Magnetic resonance imaging, Valvular disease, Magnetic resonance imaging

## Abstract

Aortic valve replacement (AVR) leads to reverse cardiac remodeling in patients with aortic stenosis (AS). The aim of this secondary pooled analysis was to assess the degree and determinants of changes in myocardial perfusion post AVR, and its link with exercise capacity, in patients with severe AS. A total of 68 patients underwent same-day echocardiography and cardiac magnetic resonance imaging with adenosine stress pre and 6–12 months post-AVR. Of these, 50 had matched perfusion data available (age 67 ± 8 years, 86% male, aortic valve peak velocity 4.38 ± 0.63 m/s, aortic valve area index 0.45 ± 0.13cm^2^/m^2^). A subgroup of 34 patients underwent a symptom-limited cardiopulmonary exercise test (CPET) to assess maximal exercise capacity (peak VO_2_). Baseline and post-AVR parameters were compared and linear regression was used to determine associations between baseline variables and change in myocardial perfusion and exercise capacity. Following AVR, stress myocardial blood flow (MBF) increased from 1.56 ± 0.52 mL/min/g to 1.80 ± 0.62 mL/min/g (*p* < 0.001), with a corresponding 15% increase in myocardial perfusion reserve (MPR) (2.04 ± 0.57 to 2.34 ± 0.68; *p* = 0.004). Increasing severity of AS, presence of late gadolinium enhancement, lower baseline stress MBF and MPR were associated with a greater improvement in MPR post-AVR. On multivariable analysis low baseline MPR was independently associated with increased MPR post-AVR. There was no significant change in peak VO_2_ post-AVR, but a significant increase in exercise duration. Change in MPR was associated with change in peak VO_2_ post AVR (r = 0.346, *p* = 0.045). Those with the most impaired stress MBF and MPR at baseline demonstrate the greatest improvements in these parameters following AVR and the magnitude of change in MPR correlated with improvement in peak VO_2_, the gold standard measure of aerobic exercise capacity.

## Background

Aortic stenosis (AS) is associated with adverse cardiac remodeling including left ventricular hypertrophy (LVH) and microvascular dysfunction. The hypertrophic response to pressure overload in severe AS is extremely variable ^1,2^. The rate of myocyte hypertrophy exceeds that of capillary angiogenesis. This leads to relatively reduced capillary density and near maximal vasodilatation to match the increased metabolic demand of the hypertrophied ventricle, resulting in increased resting myocardial blood flow (MBF). However, MBF during stress remains inadequate to meet the further increased demand during exercise, resulting in an overall reduction in myocardial perfusion reserve (MPR).

MBF can be measured non-invasively using cardiac magnetic resonance (CMR) imaging. MPR is dependent on a combination of factors including AS severity ^3^, extent of left ventricular remodelling/fibrosis ^4^ and perfusion time ^5^. MPR has been shown to be an independent predictor of exercise capacity and is inversely proportional to New York Heart Association functional class in patients with severe AS undergoing aortic valve replacement (AVR) ^4^. Impaired coronary/ myocardial flow reserve has also been shown to be an independent predictor of major adverse cardiovascular events in patients with AS ^3,6,7^.

AVR results in reverse cardiac remodeling, with left ventricular mass indexed (LVMI) regressing by 20% by 6–12 months, and the magnitude of change being greater in those with a higher LVMI at baseline ^8,9^. Improvement in MPR ^10–12^ and exercise capacity ^13^ have also been reported post-AVR, but there are limited data using quantitative CMR perfusion, and no studies with concomitant cardio-pulmonary exercise testing (CPET).

The aim of this study was to assess the determinants of change in myocardial perfusion post AVR and its link with exercise capacity, in patients with severe AS.

## Methods

This is a secondary analysis of data pooled from two prospective, observational studies of patients with severe AS(^4^ and NCT03883490) from a single tertiary referral centre. These studies were approved by the UK National Research Ethics Service (19/EM/0032) and the Local Research and Ethics committee (08/H0402/6). The study was conducted in accordance with the ethical principles outlined in the Declaration of Helsinki. All study procedures were performed in accordance with the approved study protocols, relevant guidelines and regulations. Written informed consent was obtained from each participant.

### Patient selection

Patients listed for surgical AVR (SAVR), as per clinical indication, were prospectively enrolled. Inclusion criteria were: ≥ 18 years and severe AS (defined as one of the following: aortic valve area < 1cm^2^, peak aortic velocity ≥ 4 m/s or mean pressure gradient > 40 mmHg). Exclusion criteria were other severe valve disease, atrial fibrillation, previous valve surgery, contraindication to CMR or an estimated glomerular filtration rate < 30 mL/min/1.73m^2^. Coronary artery disease (CAD) was defined as coronary artery luminal stenosis > 50%, previous myocardial infarction, coronary artery bypass graft (CABG) or percutaneous coronary intervention.

### Study procedures

All participants underwent transthoracic echocardiography and CMR (with adenosine stress perfusion if no contra-indication) at baseline and 6–12 months post-AVR. A subgroup of patients also underwent a CPET. All investigations were performed on the same day, 48 hours after discontinuing beta-blockers if having a CPET.

### Blood samples

At baseline and follow up, blood was sampled for N-terminal pro-brain natriuretic peptide (NTproBNP), and renal function.

### Echocardiography

Transthoracic echocardiography was performed using a Vivid 7 (GE Healthcare, Waukesha, Wisconsin) or iE33 (Philips Ultrasound, Netherlands) according to national guidelines ^14,15^ by an accredited sonographer. Analysis was performed offline blinded to patient details using EchoPAC software (GE Medical systems, Little Chalfont, UK) or Xcelera (Philips Ultrasound, Netherlands).

### Cardiac magnetic resonance imaging

CMR was performed using either a 1.5-T (Siemens, Avanto) or 3-T (Siemens, Skyra) scanner with retrospective ECG gating and a 6- or 18-channel phased array cardiac coil, respectively. Each participant was imaged on the same scanner using the same sequences at baseline and follow-up. Steady-state free precession cine images of the long-axis (2, 3 and 4-chamber views), aortic valve and short-axis stack of the left ventricle (LV) were acquired. First-pass perfusion images were acquired after pharmacological vasodilatation stress with adenosine, 140–210 µg/kg/min, for 3–5 min. First pass perfusion was assessed in 3 short axis slices at basal, mid-ventricular and apical levels, using a saturation recovery at 1.5 T or dual sequence T1-weighted gradient echo sequence at 3 T ^16^, with gadolinium-based contrast agent (0.05 mmol/kg at 1.5 T and 0.075 mmol/kg at 3 T) administered at 5 mL/s. Rest imaging was performed approximately 10 min after stress imaging with a further dose of contrast. A further 0.1 mmol/kg of contrast was given to those scanned at 1.5 T, to bring the total dose to 0.2 mmol/kg, whereas the total dose was 0.15 mmol/kg at 3 T. At least ten minutes following this, late gadolinium enhancement (LGE) images were acquired with the use of an inversion-recovery preparation, segmented gradient echo sequence.

All images were analysed blinded to participant details by a single observer (SA). Cardiac chambers volumetric quantification was performed using cvi42 (Version 5.10.1, Circle Cardiovascular Imaging, Calgary, Canada), with papillary muscles excluded from the LV mass quantification. Two experienced observers qualitatively assessed LGE images for focal fibrosis, categorized as present or absent, and infarct/non-infarct pattern. Right ventricular (RV) insertion point enhancement was not classed as pathological. Perfusion images were first assessed qualitatively for distribution of stress perfusion defects by two experienced observers. Quantitative analysis of myocardial blood flow was performed either by model independent deconvolution ^17^ or a machine learning approach using inline automated reconstruction and image post-processing within the Gadgetron software framework ^16^. For the latter, MBF was calculated using a blood tissue exchange model displayed on pixel-wise perfusion maps expressed in mL/min/g. Stress and rest MBF were derived for each of the 16 segments (apical segment excluded) of the American Heart Association segmentation model and averaged to calculate global MBF. Global MPR was defined as the ratio of stress to rest MBF. Microvascular dysfunction (MVD) was defined as MPR < 2.0 ^18^ in the absence of known significant epicardial CAD (> 50% luminal stenosis). Rest MBF was also normalised to the rate-pressure product (RPP), and corrected MPR was defined as the ratio of stress MBF to RPP normalised rest MBF. Additionally, total MBF was calculated as stress or rest MBF x left ventricular mass (g).

### Cardiopulmonary exercise testing

Physician supervised, symptom limited CPET was performed in a subset of participants using a bicycle ergometer. An incremental 1–min ramp protocol was used with workload increments calculated based on participant age, sex, height and weight ^19^. A 12-lead electrocardiogram was monitored continuously, and blood pressure recorded every 2 min. Expired ventilatory gases were analysed using an ErgoCard CPEX Test Station (Medisoft, Dinant, Belgium) to determine peak oxygen consumption (VO_2_). Indications for termination included limiting dyspnoea, chest discomfort or dizziness, ST segment depression of > 5 mm measured 80 ms after the J point, > 3 consecutive ventricular ectopic beats and a drop in blood pressure of > 20 mmHg from baseline or patient fatigue.

### Statistical analysis

Continuous variables were assessed for normality using graphical displays and the Shapiro–Wilk test and are expressed as mean ± standard deviation or median (interquartile range (IQR)), as appropriate. Categorical data are presented as number (percentage). Comparisons of continuous variables at baseline and follow up were performed using paired sample t-test and Wilcoxon signed rank, as appropriate, whilst Chi-squared or Fisher’s exact test were used for categorical variables. Comparisons between groups were conducted using independent samples t-test or Mann–Whitney U-test. A sensitivity analysis was conducted excluding any patients with significant coronary artery disease. The percentage change in variables from pre to post-AVR was calculated ([pre-AVR value–post-AVR value]/pre-AVR value) × 100) and used as a continuous outcome variable for linear regression using pre-AVR data as input variables. Pearson’s correlations were performed to investigate associations between baseline variables, change in myocardial perfusion and peak VO_2_. NTpro-BNP was skewed and logarithmically transformed before regression analysis. Multivariable linear regression (enter) models were constructed to determine the associations with change in MPR. Variables were considered for multivariable analysis when they were related to the dependent variable on univariate analysis with *p*-values < 0.1 or have known clinical significance. In cases of collinearity, the variable with historically the stronger prognostic importance or stronger statistical significance was chosen for the multivariable analysis. Statistical analysis was undertaken using SPSS, version 28.0 (IBM SPSS, Chicago, Illinois).

## Results

### Study population

**Fig. 1:**
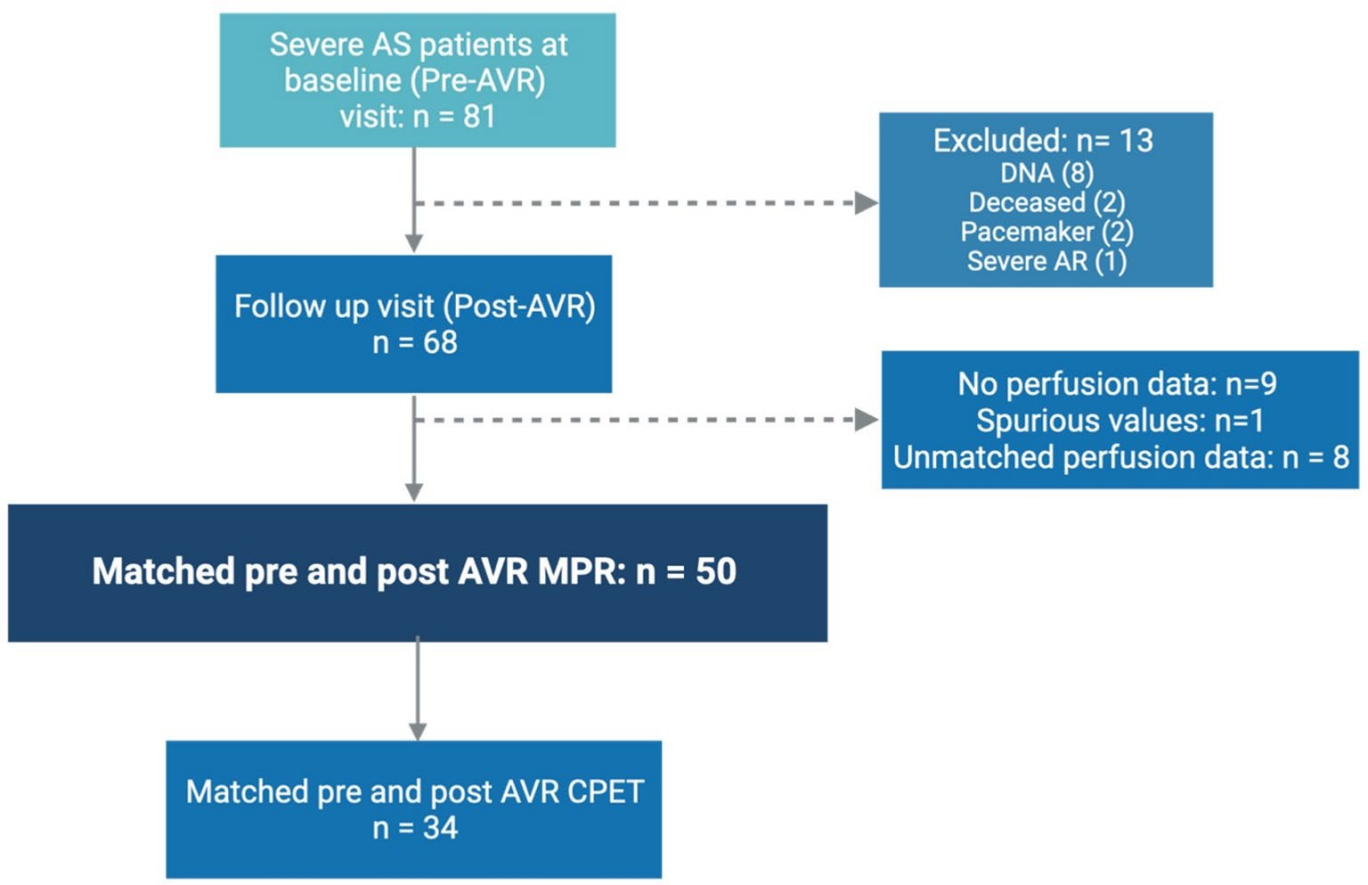
Recruitment diagram showing study population. AR, aortic regurgitation; AS, aortic stenosis; AVR, aortic valve replacement; CPET, cardiopulmonary exercise testing; DNA, did not attend; MPR, myocardial perfusion reserve.

Details of recruitment are outlined in Fig. 1. A total of 68 patients underwent assessment at a median of 193 days (IQR: 180 to 360 days) post-AVR. Matched perfusion data were available for 50 patients, of these 9 patients had CAD. Baseline characteristics are shown for these and those excluded from the main analysis in Table 1 and Supplementary Table S1, respectively. Matched pre- and post-AVR CPET data were available in 34 patients.

**Table 1:** Baseline characteristics.

Participants	50
Age (years)	67 ± 8
Male	43 (86)
BSA (m^2^)	1.98 ± 0.21
BMI (kg/m^2^)	28.9 ± 4.6
HR (bpm)	68 ± 12
SBP (mmHg)	131 ± 20
DBP (mmHg)	76 ± 9
Hypertension	32 (64)
Diabetes	10 (20)
Dyslipidaemia	13 (26)
Coronary artery disease	9 (18)
Current/Ex-smoker	33 (66)
Drug history	
ACEi/ARB	19 (38)
Beta-blocker	20 (40)
Diuretic	12 (24)
Statin	31 (62)
Bloods	
Creatinine (umol/l)	83 (73, 97)
NTproBNP (pmol/L)*	197 (74, 483)
NYHA functional class	
I	7 (14)
II	34 (68)
III	9 (18)
Echocardiographic data	
AV Vmax (m/s)	4.38 ± 0.63
AV MPG (mmHg)	46.4 ± 14.3
AVAi (cm^2^/m^2^)	0.45 ± 0.13

Values are mean ± SD, n (%) or median (interquartile range).

*n = 41.

ACEi, angiotensin converting enzyme- inhibitor; ARB, angiotensin receptor blocker; AV, aortic valve; AVAi, aortic valve area indexed to BSA; BSA, body surface area; BMI, body mass index; HR, heart rate; MPG, mean pressure gradient; NTproBNP, N terminal pro-brain natriuretic peptide; NYHA, New York Heart failure Association; SBP/DBP, systolic/ diastolic blood pressure; Vmax, peak velocity.

All but 7 participants were symptomatic (86%) at the time of AVR. The interval between baseline CMR and AVR was a median of 39 days (IQR: 22 to 62 days). Forty-nine of 50 participants received a bioprosthetic AVR, with additional procedures in 13 patients (8 CABG and 5 ascending aorta replacement).

### Cardiac remodeling post AVR

CMR parameters pre and post-AVR are shown in Table 2. Following AVR, there was a 17% reduction in LVMI and a 12% reduction in indexed LV end-diastolic volume (LVEDVi), with a 6% reduction in the mass-to-volume ratio. LV ejection fraction remained unchanged. Indexed RV end-diastolic volume decreased significantly, however RV end-systolic volume remained unchanged, resulting in a 4% reduction in the RV ejection fraction. Indexed left atrial volume (LAVI) also decreased significantly.

**Table 2:** Comparison of cardiac magnetic resonance and cardiopulmonary exercise parameters pre and post AVR.

	n	Pre-AVR	Post-AVR	% change	*p*-value
Volumes, mass and function					
LV EDVi (mL/m^2^)	50	82.9 ± 16.1	73.3 ± 12.7	−12	** < 0.001**
LV ESVi (mL/m^2^)	50	26.4 ± 9.9	23.3 ± 7.5	−12	**0.004**
LV SVi (mL/m^2^)	50	56.5 ± 11.2	50.1 ± 9.8	−11	** < 0.001**
LV EF (%)	50	68.5 ± 7.7	68.4 ± 7.0	0	0.944
LVCI (L/min/m^2^)	50	3.8 ± 0.8	3.4 ± 0.7	−10	** < 0.001**
LVMi (g/m^2^)	50	86 ± 20	71 ± 18	−17	** < 0.001**
LV mass: volume (g/mL)	50	1.04 ± 0.22	0.98 ± 0.22	−6	**0.006**
RV EDVi (mL/m^2^)	50	75.8 ± 13.5	72.0 ± 14.0	−5	**0.010**
RV ESVi (mL/m^2^)	50	28.2 ± 7.6	28.6 ± 8.8	+ 1	0.586
RV EF (%)	50	62.9 ± 6.9	60.4 ± 8.5	−4	**0.006**
LA_max_ volume indexed (mL/m^2^)	50	40.5 ± 11.5	37.0 ± 11.2	−9	**0.016**
LGE (present)	50	31 (62)	34 (68)	–	** < 0.001**
Myocardial perfusion					
Stress MBF (mL/min/g)	50	1.56 ± 0.52	1.80 ± 0.62	+ 15	** < 0.001**
Rest MBF (mL/min/g)	50	0.78 ± 0.19	0.81 ± 0.33	+ 3	0.536
Corrected rest MBF	50	0.91 ± 0.25	0.90 ± 0.46	−1	0.928
MPR	50	2.04 ± 0.57	2.34 ± 0.68	+ 15	**0.004**
Corrected MPR	50	1.79 ± 0.60	2.15 ± 0.68	+ 20	**0.001**
Cardiopulmonary exercise parameters					
Peak VO_2_ (ml/kg/min)	34	16.2 ± 4.1	16.7 ± 5.1	+ 3	0.435
% predicted peak VO_2_	34	59 (50,72)	61 (52,79)	+ 3	0.388
Peak workload (watts)	34	106 ± 35	111 ± 36	+ 4	0.089
CPET duration (s)	34	619 ± 165	643 ± 161	+ 4	**0.038**
Peak respiratory exchange ratio	34	1.11 ± 0.09	1.11 ± 0.08	−1	0.737
Peak HR (bpm)	34	133 ± 20	135 ± 18	+ 1	0.146
% predicted max HR	34	86.5 ± 11.9	88.3 ± 11.5	+ 2	0.152
Peak O_2_ pulse	34	10.3 ± 2.5	10.2 ± 2.6	0	0.957

Values are mean ± SD, n (%) or median (interquartile range). Bold *p* values are statistically significant.

Statistical tests: Comparisons were made using paired sample t-test, Wilcoxon signed rank or Chi-squared tests.

CPET, cardiopulmonary exercise test; ECV, extracellular volume fraction; EDVi, end diastolic volume indexed, ESVi, end systolic volume indexed; EF, ejection fraction; GCS, global circumferential strain; GLS, global longitudinal strain; LA, left atrium; LGE, late gadolinium enhancement; LV, left ventricle; LVCI, left ventricular cardiac index; LVMi, left ventricular mass indexed; MBF, myocardial blood flow; MPR, myocardial perfusion reserve; RPP, rate pressure product; RV, right ventricle; SVi, stroke volume indexed.

Non-ischemic fibrosis on LGE imaging was present in 28 (56%), infarct pattern in 2 (4%) and both patterns in 1 (2%) of subjects at baseline. Four patients had new areas of non-ischemic pattern LGE post-AVR.

### Myocardial perfusion

**Fig. 2:**
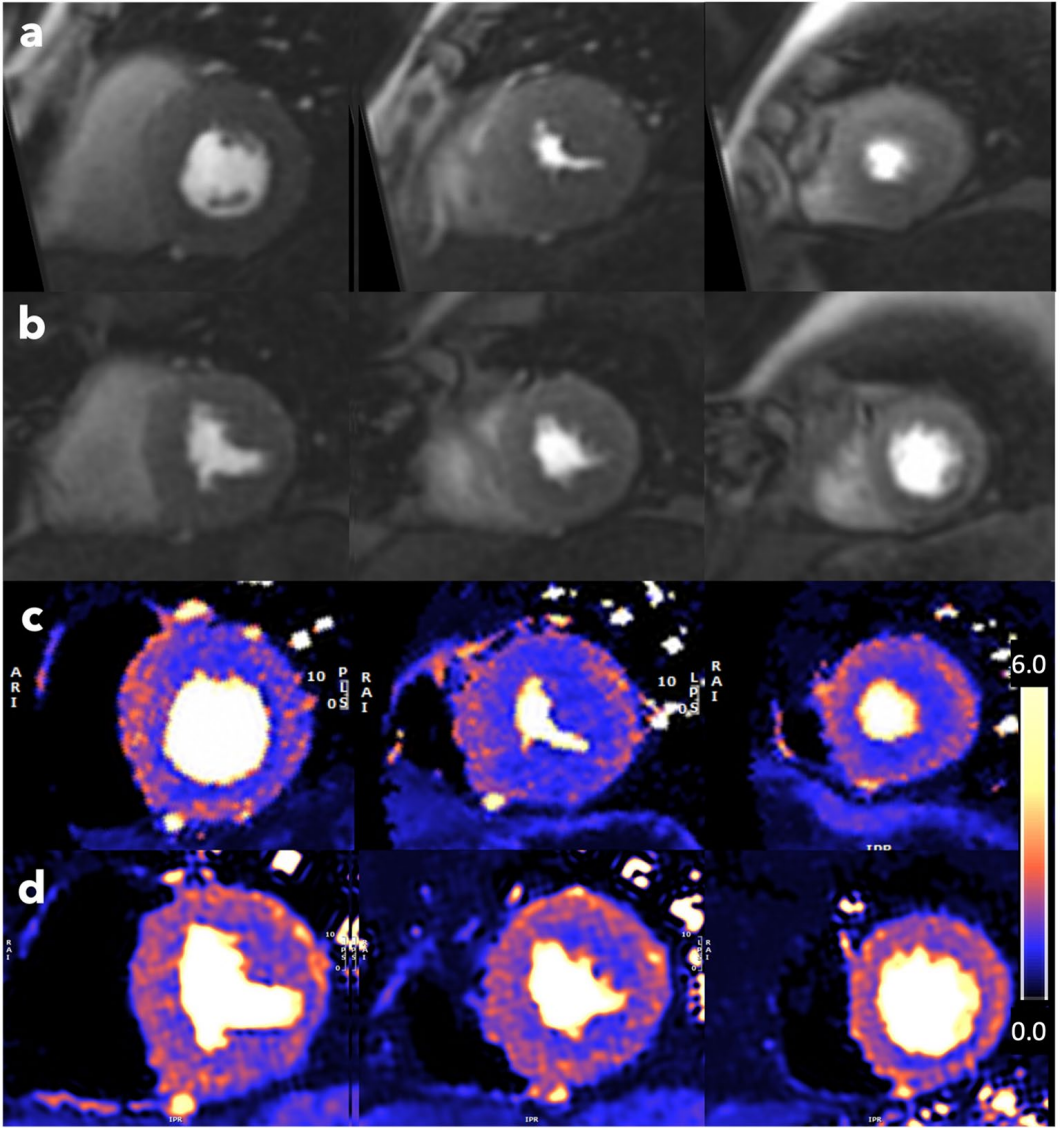
Base, mid and apical left ventricular short axis slices (left to right) for a 67-year-old male patient with severe aortic stenosis and without obstructive coronary artery disease with evidence of a global subendocardial perfusion defect suggestive of microvascular dysfunction at baseline (MPR 1.94) that improved post -AVR (MPR 3.24). (**a**) pre-AVR and (**b**) post AVR first pass stress perfusion images. (**c**) pre-AVR and (**d**) post AVR myocardial blood flow maps generated using inline automated reconstruction and post-processing showing pre-AVR stress MBF approximately 1.42 ml/min/g and post-AVR stress MBF approximately 1.73 ml/min/g. AVR, aortic valve replacement; MPR, myocardial perfusion reserve.

MVD (MPR < 2.0 in those without CAD) was present in 18 (44%) patients at baseline, and 11 (27%) patients post-AVR. Of the 10 patients with type II diabetes, 6 had MVD at baseline and 2 post-AVR. An example of pre and post AVR MBF images and maps are shown in Fig. 2. A visual regional perfusion defect was identified in 6 patients at baseline and 2 patients at follow up. There was no significant difference in MPR quantified by the two quantification techniques at baseline, though the stress and rest MBF values were higher by the model-independent deconvolution technique (Supplementary Table S2). Additionally, increasing aortic valve peak velocity and lower aortic valve area indexed were associated with lower baseline stress MBF (Supplementary Table S3) and increasing aortic valve peak velocity and mean pressure gradient with baseline MPR (Supplementary Table S4).

For the overall cohort, following AVR, there was no significant change in corrected or uncorrected rest MBF (in ml/min/g), however, stress MBF increased by 15%, with a corresponding 15–20% increase in MPR. Corresponding to the reduction in LVMI post AVR, the total rest MBF decreased from 120 (IQR: 97,160) ml/min to 97 (80,129) ml/min, *p* =  < 0.001, whilst the total stress MBF did not change significantly (257 ± 87 mL/min to 243 ± 81 mL/min, *p* = 0.180). In a sensitivity analysis after excluding those with significant CAD, similar changes in MBF and MPR were demonstrated post-AVR (Table 3).

**Table 3:** Cardiac magnetic resonance measured myocardial perfusion in patients with and without coronary artery disease.

	Pre-AVR	Post-AVR	% change	*p*-value
**AS without CAD (n = 41)**				
Stress MBF (mL/min/g)	1.68 ± 0.48	1.90 ± 0.59	+ 13	**0.003**
Rest MBF (mL/min/g)	0.82 ± 0.18	0.85 ± 0.35	+ 3	0.532
MPR	2.12 ± 0.57	2.38 ± 0.68	+ 12	**0.030**
**AS with CAD (n = 9)**				
Stress MBF (mL/min/g)	1.02 ± 0.28	1.32 ± 0.54	+ 29	0.062
Rest MBF (mL/min/g)	0.62 ± 0.14	0.62 ± 0.18	0	0.946
MPR	1.69 ± 0.45	2.15 ± 0.70	+ 27	**0.020**

Values are mean ± SD, n (%) or median (interquartile range), Bold *p* values are statistically significant.

Statistical tests: Comparisons were made using paired sample t-test.

AS, aortic stenosis; AVR, aortic valve replacement; CAD, coronary artery disease; MBF, myocardial blood flow; MPR, myocardial perfusion reserve.

Comparing patients with and without type II diabetes, although the differences in rest, stress MBF or MPR between the two groups were not statistically significant, those with diabetes had a lower MPR compared to those without (MPR 1.83 ± 0.50 *vs* 2.10 ± 0.58, *p* = 0.191). In addition, only patients without type II DM demonstrated a statistically significant improvement in stress MBF and MPR parameters post-AVR (Supplementary Table S5).

### Associations with change in stress MBF and MPR

Univariable associations with change in MPR are shown in Table 4. Increasing severity of AS parameters, presence of LGE, lower baseline stress MBF and lower baseline MPR were associated with a greater improvement in MPR post AVR. However, there were no significant associations with comorbidities including CAD, diabetes or hypertension, or baseline measures of LV remodeling (LVEDVi, LVMI, mass/volume). On multivariable analysis, low baseline MPR was the only variable to be independently associated with increase in MPR post-AVR (Table 4).

**Table 4:** Relationships between baseline clinical, imaging and blood biomarkers with change in myocardial perfusion reserve.

	Pearson’s Correlation co-efficient		Multivariable associations	
	r (95% CI)	*p*-value	B (95% CI)	*p*-value
Age	0.03 (−0.25, 0.31)	0.816		
Sex	0.13 (−0.15, 0.40)	0.356		
Hypertension	−0.13 (−0.39, 0.16)	0.372		
Diabetes	0.18 (−0.10, 0.44)	0.200	8.52 (−14.93,31.96)	0.468
CAD	0.03 (−0.25, 0.31)	0.828	−4.90 (−30.91, 21.11)	0.706
Log_10_ NTproBNP	0.10 (−0.25, 0.43)	0.569		
AV Vmax	0.34 (0.07, 0.56)	**0.016**	7.00 (−10.56, 24.47)	0.428
AV MPG	0.31 (0.03, 0.54)	**0.031**		
AVAi	−0.29 (−0.52, −0.01)	**0.043**		
LV EDVi	0.01 (−0.27, 0.28)	0.966		
LV ESVi	0.19 (−0.09, 0.45)	0.177		
LV SVi	−0.16 (−0.42, 0.12)	0.263		
LV EF	−0.25 (−0.49, 0.04)	0.087	−0.87 (−2.17, 0.42)	0.181
LVMi	−0.02 (−0.30, 0.26)	0.889		
LV mass: volume	−0.05 (−0.32, 0.24)	0.755		
LGE	0.34 (0.06, 0.56)	**0.017**	5.78 (−17.46, 29.01)	0.619
Stress MBF	−0.31 (−0.54, −0.03)	**0.029**		
Rest MBF	0.13 (−0.15, 0.40)	0.353		
MPR	−0.47 (−0.66, −0.22)	** < 0.001**	−23.23 (−43.11, −3.36)	**0.023**
	Adjusted R^2^		0.298	**0.014**

Bold *p* values are statistically significant.

Statistical tests: Pearson’s correlation to investigate associations between baseline variables and change in MPR.

Multivariable linear regression to determine the associations with change in MPR.

AV, aortic valve; AVAi, aortic valve area indexed to BSA; CAD, coronary artery disease; EDVi, end diastolic volume indexed, ESVi, end systolic volume indexed; EF, ejection fraction; LGE, late gadolinium enhancement; LV, left ventricle; LVMi, left ventricular mass indexed; MBF, myocardial blood flow; MPR, myocardial perfusion reserve; MPG, mean pressure gradient; NTproBNP, N-terminal pro-brain natriuretic peptide; SVi, stroke volume indexed; Vmax, peak velocity.

Associations with change in stress MBF are shown in Supplementary Table S6. Baseline stress MBF was inversely associated with a greater improvement in stress MBF post-AVR. However, on multi-variable analysis no significant associations were found.

### Cardiopulmonary exercise data

There were no complications during exercise testing (Table 2). Of the 34 patients with matched CPET data, 29 achieved a peak respiratory exchange ratio > 1 at baseline and 31 at follow up. Thirty-one patients achieved < 85% of their predicted peak VO_2_ at baseline and follow-up. There was no significant change in peak VO_2_ post-AVR, but a significant increase total CPET duration. Peak heart rate and peak O_2_ pulse remained unchanged.

### Correlation between change in exercise capacity and change in MPR

Percentage change in MPR was associated with percentage change in peak VO_2_ (r = 0.35, *p* = 0.045), CPET duration and peak workload (Table 5).

**Table 5:** Relationships between exercise parameters and myocardial perfusion reserve.

% change	Pearson’s correlation co-efficient	
	r (95% CI)	*p*-value
Peak workload	0.37 (0.04, 0.63)	**0.029**
CPET duration	0.38 (0.05, 0.64)	**0.027**
Peak VO_2_	0.35 (0.01, 0.61)	**0.045**

Bold *p* values are statistically significant.

Statistical test: Pearson’s correlation to investigate associations.

CPET, cardiopulmonary exercise testing; MPR, myocardial perfusion reserve; Peak VO_2_, peak oxygen consumption.

## Discussion

This is the first study to assess the effect of AVR on CMR-measured quantitative MPR and CPET assessed exercise capacity in the same cohort of patients with severe AS. In this study we have shown that a lower baseline MPR is associated with the greatest improvement in MPR post-AVR and the magnitude of this change correlated with improvement in peak VO_2_, the gold standard measure of aerobic exercise capacity.

### Myocardial perfusion

Similar to previous studies using alternative/ indirect techniques to assess measures of coronary flow reserve (CFR) ^11^ or MPR ^12^ and one recently published study using quantitative CMR measured MBF ^10^, we have shown a significant improvement in MPR post-AVR in severe AS. This was related to an improvement in stress MBF post AVR, but no change in rest MBF. The hypothesis of rest MBF maintained at ‘normal’ levels by near-maximal capillary dilatation in those with severe AS is further supported by a reduction in the total rest MBF with a reduction in LVMI post-AVR.

AS severity does not always correlate with the degree of LVH^20^ and those with inappropriate LVH experience worse outcomes ^1^. MPR has been shown to be inversely related to LVMI prior to AVR ^4^. Despite this, high LVMI at baseline was not associated with an improvement in MPR in our cohort. Following AVR, an increase in diastolic pressure/coronary filling time ^21^ and the reduction in extravascular compression caused by a reduction in intramyocardial systolic pressure ^6^ have been proposed as the main mechanisms for improvement in MBF and MPR/ CFR.

Additionally, the reduced capillary density and arteriolar remodeling ^6^ may not reverse within the first 6 months of AVR at the same rate as regression of LV mass, hence the change in MPR may not correlate with LVMI at baseline. Alterations in the myocardial microvasculature have also been demonstrated in patients with AS but without LVH ^22^, suggesting that impairment of MPR in AS is multifactorial and the mechanisms underlying its improvement post AVR are not completely understood.

Stenosis at the level of the aortic valve is anatomically below the coronary artery inflow resulting in reduced coronary perfusion pressure ^23^. Previous studies have shown greater CFR impairment with increasing severity of AS and LV remodelling ^24^. Relief of the stenosis therefore results in an acute reduction in the left ventricular end diastolic pressure (LVEDP) resulting in increased coronary perfusion ^23^. Furthermore, the augmentation in coronary flow is linearly related to aortic valve area and coronary perfusion pressure during stress/hyperaemia ^6^. Increasing AS severity results in lower coronary perfusion pressure, stress MBF and MPR and lower values are subsequently associated with greater improvements after intervention.

CAD has been reported in 40–60% of patients with AS undergoing SAVR ^25,26^ and is associated with poor outcomes ^25,27^. Current guidelines recommend performing or considering concomitant CABG in those undergoing SAVR with coronary artery stenosis ≥ 70% or ≥ 50–69% respectively ^28,29^. MBF is an integrated measure of flow through the epicardial coronary arteries and the microcirculation. In patients with CAD and AS there is a compounding effect resulting in lower values of stress and rest MBF but not MPR. Our cohort included only 9 (18%) patients with concomitant CAD and demonstrated a significant improvement in MPR at follow up similar to patients without CAD. However, the magnitude/ percentage improvement in MPR was not significantly different between these two groups.

Recent studies using CMR perfusion imaging have demonstrated reduced MPR in patients with co-existing AS and type II diabetes in comparison to isolated AS in relatively larger patient cohorts (n = 30–56) ^10,30^. Our data supports this finding, and the lack of statistical significance is likely related to the smaller sample size of only 10 patients with type II diabetes. We also showed no significant improvement in stress MBF or MPR in those with concomitant diabetes, supporting the added adverse impact of concomitant diabetes on myocardial response to AS.

Focal fibrosis as assessed by LGE has been shown to be a determinant of MPR in those with severe AS ^4^ and is irreversible post AVR ^9^. In our study, presence of LGE had a univariate association with an improvement in MPR post AVR. This association may be explained by those with greater AS severity and worse MPR at baseline (both of which were associated with improvement in MPR) having a greater LGE burden, rather than a direct link with the amount of LGE and improvement in MPR.

### Aerobic exercise capacity

Surprisingly, there was no significant improvement in peak VO_2_ post-AVR. Previous studies have also demonstrated aerobic capacity neither normalised ^31^ nor improved post-AVR on CPET ^32^ or treadmill testing ^33^ in patients with severe AS. This may be related to the post-AVR CPET being performed at 6 months, which may not be long enough to see objective improvements in exercise capacity. However, there was a significant correlation between increase in MPR and increase in exercise capacity post AVR. MPR has also previously been shown to be an independent predictor of exercise capacity in patients with severe AS ^4^. An unmatched increase in MBF in response to increased myocardial work, creates an environment vulnerable to subendocardial ischaemia and dysfunction during exercise ^34^ and, as such, provides a possible mechanism for exercise intolerance. This may also be reflective of more advanced disease, with extensive myocardial fibrosis and myocyte degeneration ^35^, which may explain the mismatch between a significant improvement in MPR but non-significant in exercise capacity. Furthermore, an exercise-echocardiography study demonstrated that patients had residual impaired myocardial function during exercise post-AVR, despite achieving similar heart rate and cardiac output to controls ^31^. A preliminary sub-analysis of the AVATAR trial (NCT02436655) has also demonstrated a non-significant deterioration in peak VO_2_ in patients with severe AS undergoing guideline-based intervention, whereas, patients undergoing early AVR demonstrated significant improvement in peak VO_2_ at 12 months post AVR ^36^. Our data are consistent with previous studies demonstrating non-significant increases in peak workload ^32^ post-AVR. It is thus possible that these changes are reflective of improvements in functional capacity.

## Clinical perspectives

Reduced MPR is common in patients with severe AS which improves significantly post AVR. Patients with the most impaired stress MBF and MPR demonstrate the greatest improvements in these parameters following AVR. This provides reassurance that the timing of aortic valve intervention in symptomatic patients with severe AS according to the current clinical guidelines is effective. Further studies are required to elucidate the mechanistic link between MPR and exercise capacity in asymptomatic patients with AS, and the effect of AVR on these, and determine if MPR has a role in risk stratification of patients for early intervention.

## Limitations

The overall study population is modest in this is single centre observational study, with only 50 patients who had matched pre and post AVR perfusion data and a smaller sub-cohort of patients that underwent CPET and therefore the results should be viewed as hypothesis generating. There is an inherent survival bias in this modest non-randomised sample as patients with more severe conditions post AVR and the deceased were not included in the analysis. However, overall, there were no significant differences in the baseline characteristics of those excluded (Supplementary Table S1). We were also limited in the number of variables that could be entered into the multivariate analysis due to the small sample size. The patients were scanned on different field strength scanners, with different imaging sequences and contrast dose. However, the same scanner, imaging sequence and contrast dose were used for pre and post-AVR scans for each individual patient, allowing comparison in pre- and post AVR parameters. Despite different perfusion imaging acquisition and analysis techniques, MPR was not significantly different between the two techniques (Supplementary Table S2). Finally, 9 (18%) participants had concomitant CAD which may alter MBF and MPR. Of these 8 underwent CABG, in whom the improvement in MPR may not be fully attributed to the SAVR. Hence a sensitivity analysis was conducted which demonstrated no significant difference in the percentage improvement in MPR, which is the main focus of this study.

## Conclusions

AVR for severe AS results in improvement in stress MBF and MPR. Lower baseline MPR was associated with the greatest improvement in MPR post AVR and the magnitude of this change correlated with change in aerobic exercise capacity.

## Supplementary Information

Supplementary Tables.

## Data Availability

The datasets used and/or analysed during the current study are available from the corresponding author on reasonable request.

## Abbreviations

AS: Aortic stenosis
AVR: Aortic valve replacement
CAD: Coronary artery disease
CMR: Cardiac magnetic resonance
CPET: Cardiopulmonary exercise test
LGE: Late gadolinium enhancement
MBF: Myocardial blood flow
MPR: Myocardial perfusion reserve
MVD: Microvascular dysfunction
VO_2_: Oxygen consumption
