# Supplementary material for: Effect of aortic valve replacement on myocardial perfusion and exercise capacity in patients with severe aortic stenosis
Source: Sci Rep. 2024 Sep 14;14:21522. doi: 10.1038/s41598-024-72480-2 (PMC11401907; doi:10.1038/s41598-024-72480-2)
Supplement: Supplementary file 1 — Supplementary Tables. [file 41598_2024_72480_MOESM1_ESM.pdf]

Supplementary

Table S1 - Baseline clinical characteristics, cardiac magnetic resonance and cardiopulmonary exercise parameters comparisons between patients in the overall cohort and those excluded in the main analysis

	Overall (n= 68)	Excluded (n= 18)	p-value
Age (years)	68 (63, 73)	70 (63, 74)	0.636
Male	52 (77)	9 (50)	**0.028**
BSA (m^2)	1.97 ± 0.21	1.94 ± 0.23	0.572
BMI (kg/m^2)	29.2 ± 4.8	30.1 ± 5.4	0.489
HR (bpm)	69 ± 13	70 ± 15	0.753
SBP (mmHg)	134 ± 22	144 ± 23	0.089
DBP (mmHg)	77 ± 9	78 ± 11	0.574
Hypertension	43 (63)	11 (61)	0.868
Diabetes	17 (25)	7 (39)	0.243
Dyslipidaemia	22 (32)	9 (50)	0.166
Coronary artery disease	10 (15)	3 (17)	0.836
Current/Ex-smoker	43 (63)	10 (56)	0.551
**Drug history**			
ACEi/ARB	25 (37)	6 (33)	0.787
Beta-blocker	28 (41)	8 (44)	0.803
Diuretic	18 (27)	6 (33)	0.564
Statin	46 (68)	15 (83)	0.192
**Bloods**			
Creatinine (umol/l)	83 (73, 97)	88 (80, 103)	0.614
NTproBNP (pmol/L)	208 (72, 510) *	251 (107, 1051)[§]	0.619
**NYHA functional class**			0.803
I	9 (13)	2 (11)	-

II	45 (66)	11 (61)	-
III	14 (21)	5 (28)	-
**Echocardiographic data**			
AV Vmax (m/s)	4.35 ± 0.62	4.29 ± 0.62	0.706
MPG (mmHg)	45.8 ± 14.3	44.2 ± 14.3	0.670
AVAi (cm^2/m^2)	0.44 ± 0.12	0.42 ± 0.11	0.489
**Volumes, mass and function**			
LV EDVi (mL/m^2)	82.0 ± 17.2	79.5 ± 20.2	0.600
LV ESVi (mL/m^2)	24.2 (19.1, 30.3)	24.6 (17.3, 29.0)	0.774
LV SVi (mL/m^2)	55.4 ± 12.3	52.2 ± 14.7	0.348
LV EF (%)	68.1 ± 9.0	66.9 ± 12.0	0.658
LVMi (g/m^2)	85.6 ± 19.7	84.6 ± 19.1	0.861
LV mass: volume (g/mL)	1.06 ± 0.23	1.09 + 0.25	0.569
RV EDVi (mL/m^2)	75.8 ± 15.3	75.8 ± 19.8	0.999
RV ESVi (mL/m^2)	29.0 ± 9.0	31.1 ± 12.2	0.401
RV EF (%)	62.0 ± 7.7	59.3 ± 9.3	0.222
LA max indexed (mL/m^2)	40.2 ± 12.3	39.3 ± 14.6	0.817

Values are mean ± SD, n (%) or median (interquartile range), **Bold** p values are statistically significant.
Statistical tests: Comparisons were made using independent samples t-test, Mann-Whitney U- test or Chi-squared.

*n= 41
§ = 7

ACEi, angiotensin converting enzyme- inhibitor; AV, aortic valve; AVAi, aortic valve area indexed to BSA, ARB, angiotensin II receptor blocker; BSA, body surface area; BMI, body mass index; EDVi, end diastolic volume indexed, ESVi, end systolic volume indexed; EF, ejection fraction; HR, heart rate; LA, left atrium; LV, left ventricle; LVMi, left ventricular mass indexed; MBF, myocardial blood flow; MPR, myocardial perfusion reserve; MPG, mean pressure gradient; NTproBNP, N terminal brain natriuretic peptide; NYHA, New York Heart failure Association; SBP/DBP, systolic/diastolic blood pressure; RV, right ventricle; SVi, stroke volume indexed; Vmax, peak velocity.

Table S2 - Differences in myocardial perfusion by quantification technique at baseline

	Optimised dual sequence	Model independent deconvolution	p-value
Stress MBF (mL/min/g)	1.21 ± 0.50	1.72 ± 0.45	**<0.001**
Rest MBF (mL/min/g)	0.61 ± 0.15	0.86 ± 0.15	**<0.001**
MPR	2.01 ± 0.70	2.06 ± 0.51	0.773

Values are mean ± SD, **Bold** p values are statistically significant.
Statistical test: Comparisons were made using independent samples t-test.

MBF, myocardial blood flow; MPR, myocardial perfusion reserve

Table S3 – Relationships between baseline aortic stenosis parameters and baseline stress myocardial blood flow

	Pearson Correlation co-efficient	
	r (95% CI)	p-value
AV Vmax	-0.35 (-0.58, -0.08)	**0.012**
AV MPG	-0.26 (-0.50, 0.02)	0.068
AVAi	0.39 (0.13, 0.60)	**0.005**

Bold p values are statistically significant.
Statistical test: Pearson's correlation to investigate associations.

AV, aortic valve; AVAi, aortic valve area indexed to BSA; MPG, mean pressure gradient

Table S4 – Relationships between baseline variables and baseline myocardial perfusion reserve

	Pearson's Correlation co-efficient	
	r (95% CI)	p-value
Age	-0.36 (-0.58, -0.09)	**0.011**
Sex	-0.17 (-0.43, 0.11)	0.239
Hypertension	-0.12 (-0.39, 0.16)	0.400
Diabetes	-0.19 (-0.44, 0.10)	0.191
Coronary artery disease	-0.30 (-0.53, -0.02)	**0.038**
Log_{10}NTproBNP	-0.43 (-0.67, -0.11)	**0.011**
AV Vmax	-0.45 (-0.65, -0.20)	**0.001**
AV MPG	-0.38 (-0.60, -0.11)	**0.006**
AVAi	0.24 (-0.04, 0.48)	0.098
LV EDVi	0.05 (-0.24, 0.32)	0.749
LV ESVi	-0.02 (-0.30, 0.26)	0.901
LV SVi	0.08 (-0.20, 0.35)	0.570
LV EF	0.06 (-0.22, 0.33)	0.687
LVMi	-0.25 (-0.49, 0.03)	0.084
LV mass: volume	-0.27 (-0.51, 0.01)	0.062
LGE	-0.40 (-0.61, -0.14)	**0.004**
Global longitudinal strain	-0.29 (-0.53, -0.001)	**0.049**
Stress MBF	0.66 (0.46, 0.79)	**<0.001**
Rest MBF	-0.21 (-0.46, 0.08)	0.147
Peak VO_2	0.42 (0.10, 0.67)	**0.013**

Bold p values are statistically significant.
Statistical test: Pearson's correlation to investigate associations.

AV, aortic valve; AVAi, aortic valve area indexed to BSA; EDVi, end diastolic volume indexed, ESVi, end systolic volume indexed; EF, ejection fraction; GLS, global longitudinal strain; LGE, late gadolinium enhancement; LV, left ventricle; LVMi, left ventricular mass indexed; MBF, myocardial blood flow; MPR, myocardial perfusion reserve; MPG, mean pressure gradient; NTproBNP, N-terminal pro-brain natriuretic peptide; SVi, stroke volume indexed.

Table S5 - Cardiac magnetic resonance measured myocardial perfusion in patients with and without type II diabetes

AS without T2D (n=40)	Pre-AVR	Post-AVR	% change	p-value
Stress MBF (mL/min/g)	1.59 ± 0.51	1.82 ± 0.62	+15	**<0.001**
Rest MBF (mL/min/g)	0.78 ± 0.18	0.82 ± 0.37	+6	0.370
MPR	2.10 ± 0.58	2.34 ± 0.67	+11	**0.024**
**AS with T2D (n= 10)**				
Stress MBF (mL/min/g)	1.44 ± 0.56	1.71 ± 0.65	+19	0.244
Rest MBF (mL/min/g)	0.79 ± 0.22	0.74 ± 0.12	-7	0.286
MPR	1.83 ± 0.50	2.33 ± 0.76	+27	0.099

Values are mean ± SD, n (%) or median (interquartile range), **Bold** p values are statistically significant.
Statistical test: Comparisons were made using paired sample t-test

T2D, type II diabetes; MBF, myocardial blood flow; MPR, myocardial perfusion reserve

Table S6 - Relationships between baseline clinical, imaging and blood biomarkers with change in stress myocardial blood flow

	Pearson's Correlation co-efficient		Multivariable associations	
	r (95% CI)	p-value	B (95% CI)	p-value
Age	-0.07 (-0.32,0.23)	0.640		
Sex	-0.06 (-0.33, 0.23)	0.694		
Hypertension	-0.07 (-0.34, 0.21)	0.636		
Diabetes	0.11 (-0.17, 0.38)	0.447		
Coronary artery disease	0.18 (-0.10, 0.44)	0.200		
Log_{10} NTproBNP	0.01 (-0.33, 0.35)	0.956		
AV Vmax	0.18 (-0.11, 0.43)	0.221		
AV MPG	0.14 (-0.14, 0.41)	0.318		
AVAi	-0.26 (-0.50, 0.02)	0.066	-58.0 (-131.0, 33.0)	0.236
LV EDVi	0.05 (-0.23, 0.33)	0.723		
LV ESVi	0.07 (-0.21, 0.34)	0.634		
LV SVi	0.01 (-0.27, 0.29)	0.924		
LV EF	-0.04 (-0.31, 0.24)	0.790		
LVMi	-0.14 (-0.40, 0.14)	0.328		
LV mass: volume	-0.22 (-0.47, 0.07)	0.130		
LA_{max} volume indexed	-0.01 (-0.28, 0.27)	0.972		
LGE	0.09 (-0.19, 0.36)	0.526		
Stress MBF	-0.28 (-0.52, -0.02)	**0.049**	-14.2 (-34.6, 6.15)	0.167
		Adjusted R^2	0.068	0.072

Bold p values are statistically significant.
Statistical tests: Pearson's correlation to investigate associations between baseline variables and change in stress MBF. Multivariable linear regression to determine the associations with change in stress MBF

AV, aortic valve; AVAi, aortic valve area indexed to BSA; EDVi, end diastolic volume indexed, ESVi, end systolic volume indexed; EF, ejection fraction; GLS, global longitudinal strain; LA, left atrium; LGE, late gadolinium enhancement; LV, left ventricle; LVMi, left ventricular mass indexed; MBF, myocardial blood flow; MPR, myocardial perfusion reserve; MPG, mean pressure gradient; NTproBNP, N-terminal pro-brain natriuretic peptide; SVi, stroke volume indexed.
